# Plasmalemmal V-ATPase as a Potential Biomarker for Lactoferrin-Based Anticancer Therapy

**DOI:** 10.3390/biom12010119

**Published:** 2022-01-12

**Authors:** Cátia Santos-Pereira, Lígia R. Rodrigues, Manuela Côrte-Real

**Affiliations:** 1 Centre of Molecular and Environmental Biology (CBMA), Department of Biology, University of Minho, 4710-057 Braga, Portugal; catiapereira@ceb.uminho.pt; 2 Centre of Biological Engineering (CEB), Department of Biological Engineering, University of Minho, 4710-057 Braga, Portugal; lrmr@deb.uminho.pt

**Keywords:** biomarker, cancer, lactoferrin, V-ATPase

## Abstract

Lactoferrin (Lf) is a milk-derived protein with well-recognized potential as a therapeutic agent against a wide variety of cancers. This natural protein exhibits health-promoting effects and has several interesting features, including its selectivity towards cancer cells, good tolerability in humans, worldwide availability, and holding a generally recognized as safe (GRAS) status. To prompt the rational clinical application of this promising anticancer compound, previous works aimed to unveil the molecular mechanisms underlying its selective anticancer activity, where plasmalemmal V-ATPase was identified as an Lf target in cancer cells. V-ATPase is a proton pump critical for cellular homeostasis that migrates to the plasma membrane of highly metastatic cancer cells contributing to the acidity of the tumor microenvironment. Cancer cells were found to be susceptible to Lf only when this proton pump is present at the plasma membrane. Plasmalemmal V-ATPase can thus be an excellent biomarker for driving treatment decisions and forecasting clinical outcomes of Lf-based anticancer strategies. Future research endeavors should thus seek to validate this biomarker by thorough preclinical and clinical studies, as well as to develop effective methods for its detection under clinical settings.

## 1. Introduction

Cancer is one of the leading causes of death worldwide, which accounted for nearly 10 million deaths in 2020, according to the World Health Organization. It describes a group of diseases characterized by the rapid and uncontrolled growth of abnormal cells, which can then invade and spread to other organs [1]. Cancer development is a complex and multistep process during which cancer cells undergo a series of changes that are known as the hallmarks of cancer, including evasion of cell death and immune response, energy metabolism reprogramming, creation of a distinctive tumor microenvironment, and angiogenesis, among others [2]. Despite all the advances and research throughout the years, cancer treatment remains a tremendous challenge, and effective treatment strategies are still missing for the vast majority of cancers [3]. New and improved anticancer approaches are thus needed to tackle this major public health concern, reduce the global cancer burden, and improve cancer patients’ survival overall.

Naturally occurring compounds have a major role in cancer treatment and prevention. They are a great source of anticancer agents, which promote the natural immune system, and have reduced toxicity, side effects, and drug resistance risk [4]. Indeed, many anticancer drugs derived from natural sources are already on the market [5]. Milk and colostrum are one of the sources of natural anticancer compounds, in particular of the lactoferrin (Lf) protein.

## 2. More Than Just a Milk-Derived Protein: Anticancer Activity of Lactoferrin

Lf is an iron-binding glycoprotein that exhibits multiple and diverse biological activities, which has inspired generations of researchers devoted to uncovering its mechanisms of action towards developing innovative Lf-based applications. It is secreted into different fluids of mammalian species, including saliva, sweat, tears, gastrointestinal fluids, and semen, among others. Lf synthesis can be constitutive at the mucosal surfaces, hormone-dependent in the case of the genital tract or mammary gland, or even occur at well-defined stages of cell differentiation, namely via neutrophils during their differentiation process [6]. However, though the concentration is extremely dependent on the lactation stage, milk and colostrum are by far the most abundant sources of Lf [7]. Lfs from different species (e.g., human, bovine, camel, mouse, and goat) have been investigated regarding their multifunctional roles, and their anticancer activity particularly stands out.

The anticancer activity of Lf was unraveled in the mid 90′s after findings showed that the whey fraction of bovine milk [8] and human Lf (hLf) [9] inhibit tumor growth and metastasis in rats and mice, respectively. Since then, a myriad of studies have demonstrated the anticancer effects of Lf in a broad range of cancer types, including breast [10], lung [11], leukemia [12], melanoma [13], osteosarcoma, and prostate [14], among others. New insights concerning the anticancer role of Lf arose when the Lf gene expression was found to be negatively associated with cancer progression and metastasis [15,16] and positively associated with patient life expectancy [17]. Indeed, the Lf gene (*LTF*) is downregulated in many types of cancer cells in comparison with their normal counterparts [18], and its overexpression inhibits the proliferation of cancer cells [19]. Thus, Lf has been suggested to act as a tumor suppressor gene [15,19]. Accordingly, in a recent study, *LTF* deficiency in mice was shown to promote the metastatization of melanoma cells to lungs, as compared to *LTF* +/+ mice [20].

Some of the research on Lf anticancer activity was already translated to the clinics, and several clinical trials have been performed with encouraging results. Kozu et al. reported that 1-year daily oral intake of 3 g of bovine Lf (bLf) is efficient in delaying the growth of colorectal polyps in participants less than 64 years old, and bLf intake was suggested as a good adjuvant for colorectal polyp extraction [21]. The combination of recombinant human Lf (rhLf) with carboplatin and paclitaxel rendered an overall patient survival improvement in patients with non-small cell lung cancer (NSCLC) [22]. The rhLf (talactoferrin) was further tested in patients with metastatic renal carcinoma and NSCLC who had previously been subjected to conventional chemotherapy, where encouraging anticancer effects were detected, though in some cases the response rate endpoint was not met, likely because the enrolled patients were heavily pretreated [23,24]. Lf is thus a promising natural dietary protein with great potential for anticancer therapy.

## 3. A Box Full of Surprises

When we consider its anticancer activity and its proven advantages, Lf is a “box” full of good surprises. On top of them is its selectivity. Indeed, at identical concentrations, Lf was shown to be cytotoxic to cancer cells while having no effect on their non-cancer cell counterparts in several in vitro studies [14,25,26]. Importantly, Lf was also well-tolerated in the clinical trials, with no serious side-effects being observed, while signs of immunomodulation were detected [21,22,23,24]. Moreover, when given in combination with chemotherapeutics, bLf was found to minimize side effects such as anemia and mucositis [27]. Accordingly, both the European Food Safety Authority (EFSA) [28] and the US Food and Drug Administration (FDA) [29] have recognized Lf as safe for various applications. As such, different Lf-containing commercial products are now available either with Lf alone or in combination with probiotics or supplements, such as infant formula, prebiotic foods, yogurts, pet food, skin and oral care products, and sports supplements [30]. Moreover, due to its easy availability and inexpensive production from milk, it is a low-cost protein available worldwide through different companies [30]. As a curiosity, Lf is one of the few proteins with its own dedicated congress (International Conference on Lactoferrin), highlighting its importance as a multifunctional macromolecule.

Combining all these advantages, Lf has the desirable features to be successfully applied in cancer therapy, and the studies of their mechanisms of action can provide important clues for its rational and targeted application. Indeed, some mechanisms of action have been proposed to underly Lf anticancer activity such as cell cycle arrest, apoptosis, and ferroptosis induction (triggered by low iron-saturated and saturated form, respectively), immunomodulation against cancer, inhibition of vascular endothelial growth factor (VEGF)-mediated angiogenesis, and reversion of epithelium-to-mesenchymal transition leading to metastasis inhibition [31,32,33,34,35,36,37,38,39]. More recently, in-depth mechanistic studies have been conducted to understand Lf selectivity towards cancer cells and identify its molecular targets.

## 4. Interaction of Lactoferrin with V-ATPase

Finding the most susceptible cancer types to Lf, as well as its targets and mechanisms of action in cancer cells is of paramount importance to boost the clinical effectiveness of Lf. In this line, bLf was found to be selectively cytotoxic to highly metastatic cancer cells in comparison with lowly metastatic and non-tumorigenic cell lines [14,25]. The higher Lf cytotoxicity against highly metastatic cancer cells was associated with their higher extracellular acidification rate and intrinsic differences in their V-ATPase expression levels and localization.

V-ATPases are ATP-driven proton pumps essential for maintaining cellular homeostasis and for a myriad of cellular functions, including intracellular trafficking, nutrient uptake, hormone maturation, and bone remodeling, among others. A collection of studies revealed the preponderant roles of these proton pumps in cancer. Indeed, V-ATPases are known to migrate to the plasma membrane of cancer cells contributing to the acidification of the tumor microenvironment, which in turn promotes the activity of metalloproteinases and other enzymes responsible for extracellular matrix degradation, contributing to invasion, migration, and metastasis of cancer cells [40].

In light of this evidence, bLf selectivity was shown to be directly associated with the plasma membrane localization of V-ATPase and higher expression levels in highly metastatic cancer cells derived from breast and prostate cancer, as well as osteosarcoma. Indeed, the lowly metastatic cancer and non-tumorigenic cells, which exhibit only intracellular V-ATPase at lower expression levels, were resistant to bLf. In agreement, bLf was shown to inhibit cell proliferation, induce cell death through apoptosis, impair extracellular acidification rate, and induce intracellular acidification only in the highly metastatic cancer cells. It is noteworthy that the latter events are both known to depend on V-ATPase function. Further evidence on the effect of bLf on V-ATPase activity was demonstrated in purified rat liver lysosomes, which are enriched in this proton pump, where bLf was shown to inhibit both the proton pumping and ATP hydrolytic activities of V-ATPase. The effect of bLf on intracellular V-ATPases was also assessed, where it was shown to induce lysosomal alkalinization selectively in the highly metastatic cancer cells. It is worth mentioning that V-ATPase is the major contributor for the acidic lysosomal pH maintenance [14,25].

All in all, these works demonstrated that V-ATPase is a molecular target of bLf in cancer cells, and that its overexpression and presence at the plasma membrane are critical for bLf anticancer activity. In this light, Lf would be effective against cancer cells in which V-ATPase has been found at the plasma membrane. A recent work that reviewed the types of cancer cells harboring plasmalemmal V-ATPase identified cell lines and tissues derived from melanoma, breast, musculoskeletal, pancreatic, prostate, liver, lung, ovarian and esophageal cancer as having this proton pump at the plasma membrane, in contrast with their non-cancer or less invasive counterparts [40]. These types of cancer cells are those where the odds of success of Lf-based treatment would be higher.

Recently, a computational approach was developed to predict how Lf and V-ATPase interact. Atomistic models of different V-ATPase-Lf complexes (predicted by molecular docking) were generated and further refined through free binding energy calculations to estimate their binding affinities. Data suggested that Lf binds in the ATP hydrolysis sites of V-ATPase, blocking ATP access, which will primarily inhibit ATP hydrolysis and, consequently, the activity of the whole proton pump. Key binding residues were further identified as critical for V-ATPase-Lf interaction, from which a set of amino acids located at the highly cationic N-terminal region of Lf stood out [41]. It is worth mentioning that further experimental research must be conducted to validate the proposed binding mechanism and key residues identified.

## 5. V-ATPase as a Potential Biomarker for Lactoferrin-Based Anticancer Strategies

The finding of biomarkers has revolutionized and redefined therapeutic strategies against cancer. Biomarkers are particular biological, biochemical, or physical features that can have different clinical applications in oncology, including evaluation of disease progression and recurrence, diagnosis/prognosis determination, prediction of treatment response, and assessment of cancer development risk [42,43]. The knowledge provided by discovering cancer biomarkers has contributed to the development of novel targeting agents with great potential for cancer therapy. Indeed, applying this knowledge to clinical practice has been successful in improving clinical outcomes and avoiding/reducing off-target effects and associated toxicity [44,45,46]. Biomarkers have thus become a major breakthrough in cancer therapy, and their continued search is crucial for developing new and improved cancer therapies and moving towards personalized medicine.

**Figure 1:**
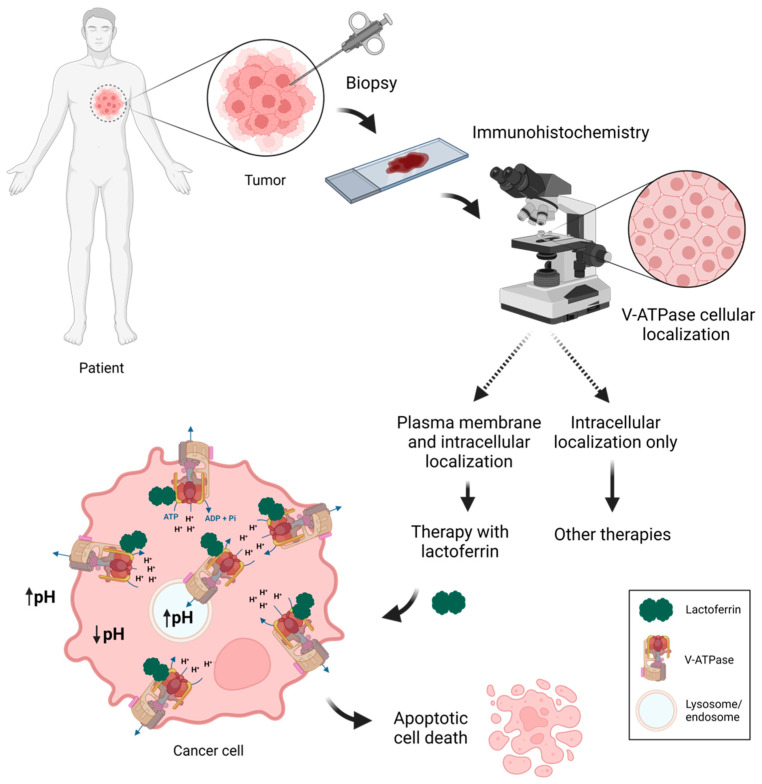
Plasmalemmal V-ATPase as a potential biomarker for guiding treatment decisions regarding Lf-based anticancer strategies. Tumor biopsies followed by immunohistochemistry with anti-V-ATPase antibodies would allow determining V-ATPase cellular localization. If it is at the plasma membrane, then Lf treatment would be effective as the protein would inhibit V-ATPase causing intracellular acidification and extracellular and lysosomal alkalinization, ultimately leading to apoptotic cell death of cancer cells. If V-ATPase is only localized intracellularly, then Lf treatment would not be successful and other therapeutic interventions should be considered. Created with BioRender.com.

In this line, knowing the molecular targets of a particular drug is critical for defining patients who would benefit from a therapeutic intervention based on it, and for performing a rational design of preclinical and clinical studies. In light of the knowledge herein presented regarding the mechanisms underlying the anticancer activity of Lf, plasmalemmal V-ATPase can be considered a biomarker for predicting response to Lf treatment and for guiding treatment decisions. In fact, if the tumor cells exhibit V-ATPase at the plasma membrane, Lf treatment would be effective as it would inhibit the proton pump and trigger the subsequent cascade of molecular events that culminate in cancer cell death. In contrast, if V-ATPase is only localized intracellularly, the tumor cells would be resistant to Lf treatment, as shown for the lowly metastatic and non-cancer cells. In addition to the cancer cell types where this has been validated (osteosarcoma, breast, and prostate cancer), other cancer cells where V-ATPase was found at the plasma membrane, such as derived from lung, pancreatic and ovarian cancer may be cancer types promising for Lf-based therapy. Therefore, a potentially good approach for Lf-based anticancer strategies would be to perform cancer biopsies, followed by immunohistochemistry to find V-ATPase localization, before deciding whether to use Lf as part of the therapeutic intervention (Figure 1). This may also be applied when Lf is used as an adjuvant with other chemotherapeutics.

Though this pipeline has good potential, we cannot forget that this line of research is still in its infancy, and thorough in vivo studies and clinical trials must be performed to validate plasmalemmal V-ATPase as a biomarker for Lf-based anticancer therapies. Indeed, although many cancer biomarkers are being identified by R&D activities, only a few are transferred to the clinics. Thus, future research avenues may be focused on the clinical validation and evaluation of the clinical utility of plasmalemmal V-ATPase for Lf therapies. Moreover, it would be interesting to explore the potential of V-ATPase as a biomarker in other types of cancer cells, not only in those where V-ATPase is already known to be at the plasma membrane but also in other cancer cell types in which the V-ATPase localization is not known. In particular, since the majority of clinical trials on the anticancer activity of Lf have been conducted in patients with lung and colorectal cancer [22,23,24], ascertaining whether this proton pump is a reliable biomarker for Lf treatment decisions in these two cancer cell types would contribute to validate this possibility. Technical aspects should also not be forgotten, as employing a robust assay able to perform this kind of detailed cellular analysis in a setting compatible with clinical labs may also be a challenge that requires future research endeavors.

When considering a therapeutic application of Lf, other important aspects must be taken into account, including the route of administration, its bioavailability, and the ability of the protein to reach the tumor site. The most convenient and used method is oral administration, although there are contradictory studies regarding protein degradation, which if extensive will cause low absorption and low bioavailability [47]. Indeed, studies in humans have shown that rhLf produced by *Aspergillus awamori* is digested in the upper gastrointestinal (GI) tract, not reaching the colon [48], while 64–79% of bLf (depending on its iron saturation state) was shown to enter the intestine in the intact form [49]. bLf seems, therefore, to be more resistant to the degradation in the upper GI tract than rhLf, and it is suggested that its oral administration should be performed before meals to avoid its degradation induced by the more acidic pH upon digestion, hence increasing its bioavailability [50]. Though still controversial, once at the intestine, it is believed that a portion of Lf will interact with its receptors in the enterocytes and be transferred to the bloodstream and the target tissues [50].

However, considering its known low bioavailability [47], whether oral administration of Lf (either from human or bovine origin) will be successful in promoting its delivery to the tumor site and, consequently, its interaction with V-ATPase is still an important open question that should be pursued to validate V-ATPase as a biomarker and to boost Lf use in therapies. Hence, the next step would be to study whether Lf given orally can reach the tumor sites to interact with V-ATPase. A highly interesting fact is that, owing to its tumor-targeting ability, Lf has been used to functionalize the surface of formulations containing other anticancer compounds aiming to promote active targeting to the tumor (e.g., [51,52,53]). We can thus hypothesize that oral Lf may reach the tumor by itself. Nevertheless, other mechanisms similar to those reported by Kruzel et al. may be involved. Indeed, inspired by the fact that despite Lf having low bioavailability, it induces systemic effects, the authors conducted experimental research aimed at understanding the underlying mechanisms. The effect of rhLf produced in Chinese Hamster Ovary (CHO) cells, administered either orally or intravenously, in gene expression of blood cells was evaluated in rats. They concluded that the majority of genes whose expression was altered by oral rhLf were also modified by the intravenous treatment, which leads to similar biological activities independent of the administration route. The observed modifications in gene expression were suggested to be due to perturbation of signal transduction mechanisms, namely those involved in cytokine and inflammatory response pathways, as well as oxidative stress pathways [47]. Given that V-ATPase is also regulated by different signaling pathways such as PI3K/AKT/mTOR and cAMP/PKA pathways [54], there is also a possibility that Lf inhibits V-ATPase activity indirectly by changing signal transduction.

Other strategies to prompt Lf interaction with V-ATPase and an effective Lf anticancer activity may also be explored. Targeted delivery systems of Lf may be an excellent hypothesis to promote Lf-V-ATPase interaction. In fact, microencapsulation using several different technologies has been shown to improve Lf bioavailability by enhancing its gastro-protection, to promote its effective targeting to the tumor cells, and to potentiate its anticancer activities [55,56,57,58]. Intratumoral injection of Lf may also be an efficient way to deliver Lf directly into the tumor and boost its interaction with V-ATPase. However, in this case, only hLf could be used since, due to compatibility issues, bLf can only be enterally delivered. Indeed, though they share a high percentage of homology [59], bLf and hLf have distinct glycosylation patterns [60]. A good alternative may be a rhLf produced in CHO cells that greatly resembles the glycan profile of hLf, being less immunogenic and consequently suitable for parenteral use [61] while maintaining Lf biological activities [62].

## 6. Conclusions

The search for the mechanisms of action of a given drug or compound is of utmost importance for discovering targets and biomarkers, which in turn helps to effectively select patients more prone to respond to the drug treatment. Plasmalemmal V-ATPase was identified as a critical molecular target of Lf in cancer cells. When this proton pump is absent from the plasma membrane, cancer cells are not affected by Lf, making it an ineffective treatment. Plasmalemmal V-ATPase can thus be a promising biomarker for driving Lf treatment decisions and forecasting clinical outcomes. However, a long path has yet to be trod to validate plasmalemmal V-ATPase as a biomarker for Lf-based therapy, namely regarding its validation through in vivo studies and in rational clinical trials, the identification of the cancer types where Lf could be applied, the determination of administration route, the optimization of the dose and frequency, and the development of an effective high-throughput method for the detection of V-ATPase at the plasma membrane of cancer cells in clinical settings. If the candidate biomarker plasmalemmal V-ATPase surpasses all these hurdles and validation steps, it should lead to a tailored application of Lf in cancer therapy, paving the way to precision medicine that will likely prompt clinically effective outcomes.
